# Supplementary material for: The stigma associated with cutaneous leishmaniasis (CL) and mucocutaneous leishmaniasis (MCL): A protocol for a systematic review
Source: PLoS One. 2023 May 11;18(5):e0285663. doi: 10.1371/journal.pone.0285663 (PMC10174477; doi:10.1371/journal.pone.0285663)
Supplement: S2 File — (PDF) [file pone.0285663.s002.pdf]

Search number	Query	Filters	Results
1	((((((((((((((((((((((((((((((((Dermal leishman*[Title/Abstract]) OR (cutaneous leishman*[Title/Abstract])) OR (oriental sore[Title/Abstract])) OR (Uta[Title/Abstract])) OR (Chiclero ulcer[Title/Abstract])) OR (tropical sore[Title/Abstract])) OR (Bagdad boil[Title/Abstract])) OR (Baghdad boil[Title/Abstract])) OR (Bauer ulcer[Title/Abstract])) OR (Delhi boil[Title/Abstract])) OR (Aleppo boil[Title/Abstract])) OR (Aleppo button[Title/Abstract])) OR (Jericho boil[Title/Abstract])) OR (one year sore[Title/Abstract])) OR (one year ulcer[Title/Abstract])) OR (tegumentary leishmaniasis[Title/Abstract])) OR (Biskra button[Title/Abstract])) OR (Biskra nodule[Title/Abstract])) OR (Calcutta ulcer[Title/Abstract])) OR (Jericho button[Title/Abstract])) OR (Kandahar sore[Title/Abstract])) OR (Lahore sore[Title/Abstract])) OR (Oriental button[Title/Abstract])) OR (Pian bois[Title/Abstract])) OR (Old World leishmaniasis[Title/Abstract])) OR (Mucosal Leishman*[Title/Abstract])) OR (Mucocutaneous Leishman*[Title/Abstract])) OR (muco cutaneous Leishman*[Title/Abstract])) OR (espundia[Title/Abstract])) OR (nasopharyngeal Leishman*[Title/Abstract])) OR (New World leishmaniasis[Title/Abstract])) OR (American leishmaniasis[Title/Abstract])) OR (leishmaniasis americana[Title/Abstract])		14,815
2	((((((((((((((stigma*[Title/Abstract]) OR (discriminat*[Title/Abstract])) OR (stereotyp*[Title/Abstract])) OR ("negative attitude"[Title/Abstract])) OR ("psychological"[Title/Abstract])) OR ("psychosocial"[Title/Abstract])) OR ("social consequences"[Title/Abstract])) OR (scar*[Title/Abstract])) OR (disfigur*[Title/Abstract])) OR (self-stigma[Title/Abstract])) OR ("label avoidance"[Title/Abstract])) OR ("disgrace"[Title/Abstract])) OR ("shame"[Title/Abstract])) OR ("perception"[Title/Abstract])) OR ("rejection"[Title/Abstract])		1,203,677

3	(((((((((((((((((((((((((((((((((Dermal leishman*[Title/Abstract]) OR (cutaneous leishman*[Title/Abstract])) OR (oriental sore[Title/Abstract])) OR (Uta[Title/Abstract])) OR (Chiclero ulcer[Title/Abstract])) OR (tropical sore[Title/Abstract])) OR (Bagdad boil[Title/Abstract])) OR (Baghdad boil[Title/Abstract])) OR (Bauer ulcer[Title/Abstract])) OR (Delhi boil[Title/Abstract])) OR (Aleppo boil[Title/Abstract])) OR (Aleppo button[Title/Abstract])) OR (Jericho boil[Title/Abstract])) OR (one year sore[Title/Abstract])) OR (one year ulcer[Title/Abstract])) OR (tegumentary leishmaniasis[Title/Abstract])) OR (Biskra button[Title/Abstract])) OR (Biskra nodule[Title/Abstract])) OR (Calcutta ulcer[Title/Abstract])) OR (Jericho button[Title/Abstract])) OR (Kandahar sore[Title/Abstract])) OR (Lahore sore[Title/Abstract])) OR (Oriental button[Title/Abstract])) OR (Pian bois[Title/Abstract])) OR (Old World leishmaniasis[Title/Abstract])) OR (Mucosal Leishman*[Title/Abstract])) OR (Mucocutaneous Leishman*[Title/Abstract])) OR (muco cutaneous Leishman*[Title/Abstract])) OR (espundia[Title/Abstract])) OR (nasopharyngeal Leishman*[Title/Abstract])) OR (New World leishmaniasis[Title/Abstract])) OR (American leishmaniasis[Title/Abstract])) OR (leishmaniasis americana[Title/Abstract])) AND (((((((((((((((stigma*[Title/Abstract]) OR (discriminat*[Title/Abstract])) OR (stereotyp*[Title/Abstract])) OR ("negative attitude"[Title/Abstract])) OR ("psychological"[Title/Abstract])) OR ("psychosocial"[Title/Abstract])) OR ("social consequences"[Title/Abstract])) OR (scar*[Title/Abstract])) OR (disfigur*[Title/Abstract])) OR (self-stigma[Title/Abstract])) OR ("label avoidance"[Title/Abstract])) OR ("disgrace"[Title/Abstract])) OR ("shame"[Title/Abstract])) OR ("perception"[Title/Abstract])) OR ("rejection"[Title/Abstract]))		867

4	(((((((((((((((((((((((((((((((((Dermal leishman*[Title/Abstract]) OR (cutaneous leishman*[Title/Abstract])) OR (oriental sore[Title/Abstract])) OR (Uta[Title/Abstract])) OR (Chiclero ulcer[Title/Abstract])) OR (tropical sore[Title/Abstract])) OR (Bagdad boil[Title/Abstract])) OR (Baghdad boil[Title/Abstract])) OR (Bauer ulcer[Title/Abstract])) OR (Delhi boil[Title/Abstract])) OR (Aleppo boil[Title/Abstract])) OR (Aleppo button[Title/Abstract])) OR (Jericho boil[Title/Abstract])) OR (one year sore[Title/Abstract])) OR (one year ulcer[Title/Abstract])) OR (tegumentary leishmaniasis[Title/Abstract])) OR (Biskra button[Title/Abstract])) OR (Biskra nodule[Title/Abstract])) OR (Calcutta ulcer[Title/Abstract])) OR (Jericho button[Title/Abstract])) OR (Kandahar sore[Title/Abstract])) OR (Lahore sore[Title/Abstract])) OR (Oriental button[Title/Abstract])) OR (Pian bois[Title/Abstract])) OR (Old World leishmaniasis[Title/Abstract])) OR (Mucosal Leishman*[Title/Abstract])) OR (Mucocutaneous Leishman*[Title/Abstract])) OR (muco cutaneous Leishman*[Title/Abstract])) OR (espundia[Title/Abstract])) OR (nasopharyngeal Leishman*[Title/Abstract])) OR (New World leishmaniasis[Title/Abstract])) OR (American leishmaniasis[Title/Abstract])) OR (leishmaniasis americana[Title/Abstract])) AND (((((((((((((((stigma*[Title/Abstract]) OR (discriminat*[Title/Abstract])) OR (stereotyp*[Title/Abstract])) OR ("negative attitude"[Title/Abstract])) OR ("psychological"[Title/Abstract])) OR ("psychosocial"[Title/Abstract])) OR ("social consequences"[Title/Abstract])) OR (scar*[Title/Abstract])) OR (disfigur*[Title/Abstract])) OR (self-stigma[Title/Abstract])) OR ("label avoidance"[Title/Abstract])) OR ("disgrace"[Title/Abstract])) OR ("shame"[Title/Abstract])) OR ("perception"[Title/Abstract])) OR ("rejection"[Title/Abstract]))	English	771

5	(((((((((((((((((((((((((((((((((Dermal leishman*[Title/Abstract]) OR (cutaneous leishman*[Title/Abstract])) OR (oriental sore[Title/Abstract])) OR (Uta[Title/Abstract])) OR (Chiclero ulcer[Title/Abstract])) OR (tropical sore[Title/Abstract])) OR (Bagdad boil[Title/Abstract])) OR (Baghdad boil[Title/Abstract])) OR (Bauer ulcer[Title/Abstract])) OR (Delhi boil[Title/Abstract])) OR (Aleppo boil[Title/Abstract])) OR (Aleppo button[Title/Abstract])) OR (Jericho boil[Title/Abstract])) OR (one year sore[Title/Abstract])) OR (one year ulcer[Title/Abstract])) OR (tegumentary leishmaniasis[Title/Abstract])) OR (Biskra button[Title/Abstract])) OR (Biskra nodule[Title/Abstract])) OR (Calcutta ulcer[Title/Abstract])) OR (Jericho button[Title/Abstract])) OR (Kandahar sore[Title/Abstract])) OR (Lahore sore[Title/Abstract])) OR (Oriental button[Title/Abstract])) OR (Pian bois[Title/Abstract])) OR (Old World leishmaniasis[Title/Abstract])) OR (Mucosal Leishman*[Title/Abstract])) OR (Mucocutaneous Leishman*[Title/Abstract])) OR (muco cutaneous Leishman*[Title/Abstract])) OR (espundia[Title/Abstract])) OR (nasopharyngeal Leishman*[Title/Abstract])) OR (New World leishmaniasis[Title/Abstract])) OR (American leishmaniasis[Title/Abstract])) OR (leishmaniasis americana[Title/Abstract])) AND (((((((((((((((stigma*[Title/Abstract]) OR (discriminat*[Title/Abstract])) OR (stereotyp*[Title/Abstract])) OR ("negative attitude"[Title/Abstract])) OR ("psychological"[Title/Abstract])) OR ("psychosocial"[Title/Abstract])) OR ("social consequences"[Title/Abstract])) OR (scar*[Title/Abstract])) OR (disfigur*[Title/Abstract])) OR (self-stigma[Title/Abstract])) OR ("label avoidance"[Title/Abstract])) OR ("disgrace"[Title/Abstract])) OR ("shame"[Title/Abstract])) OR ("perception"[Title/Abstract])) OR ("rejection"[Title/Abstract]))	English, Portuguese	784

6	(((((((((((((((((((((((((((((((((Dermal leishman*[Title/Abstract]) OR (cutaneous leishman*[Title/Abstract])) OR (oriental sore[Title/Abstract])) OR (Uta[Title/Abstract])) OR (Chiclero ulcer[Title/Abstract])) OR (tropical sore[Title/Abstract])) OR (Bagdad boil[Title/Abstract])) OR (Baghdad boil[Title/Abstract])) OR (Bauer ulcer[Title/Abstract])) OR (Delhi boil[Title/Abstract])) OR (Aleppo boil[Title/Abstract])) OR (Aleppo button[Title/Abstract])) OR (Jericho boil[Title/Abstract])) OR (one year sore[Title/Abstract])) OR (one year ulcer[Title/Abstract])) OR (tegumentary leishmaniasis[Title/Abstract])) OR (Biskra button[Title/Abstract])) OR (Biskra nodule[Title/Abstract])) OR (Calcutta ulcer[Title/Abstract])) OR (Jericho button[Title/Abstract])) OR (Kandahar sore[Title/Abstract])) OR (Lahore sore[Title/Abstract])) OR (Oriental button[Title/Abstract])) OR (Pian bois[Title/Abstract])) OR (Old World leishmaniasis[Title/Abstract])) OR (Mucosal Leishman*[Title/Abstract])) OR (Mucocutaneous Leishman*[Title/Abstract])) OR (muco cutaneous Leishman*[Title/Abstract])) OR (espundia[Title/Abstract])) OR (nasopharyngeal Leishman*[Title/Abstract])) OR (New World leishmaniasis[Title/Abstract])) OR (American leishmaniasis[Title/Abstract])) OR (leishmaniasis americana[Title/Abstract])) AND (((((((((((((((stigma*[Title/Abstract]) OR (discriminat*[Title/Abstract])) OR (stereotyp*[Title/Abstract])) OR ("negative attitude"[Title/Abstract])) OR ("psychological"[Title/Abstract])) OR ("psychosocial"[Title/Abstract])) OR ("social consequences"[Title/Abstract])) OR (scar*[Title/Abstract])) OR (disfigur*[Title/Abstract])) OR (self-stigma[Title/Abstract])) OR ("label avoidance"[Title/Abstract])) OR ("disgrace"[Title/Abstract])) OR ("shame"[Title/Abstract])) OR ("perception"[Title/Abstract])) OR ("rejection"[Title/Abstract]))	English, Portuguese, Spanish	796
