# Supplementary material for: The stigma associated with cutaneous leishmaniasis (CL) and mucocutaneous leishmaniasis (MCL): A protocol for a systematic review
Source: PLoS One. 2023 May 11;18(5):e0285663. doi: 10.1371/journal.pone.0285663 (PMC10174477; doi:10.1371/journal.pone.0285663)
Supplement: S3 File — (PDF) [file pone.0285663.s003.pdf]

S2 File. Form to extract data of included studies

Reviewer –
Date –

Id number	
**Study details**	
English title	
Original title (If any)	
Doi	
Pubmed id	
Main author	
Publication year	
Language	
Database	
Journal	
**Study characteristics**	
Study design	
Study objectives	
Study setting	
Time period of the research	

**Demographics of participants**	
Age	
Gender	
Ethnicity	
Socio economic status	
Education levels	
Occupation	
Income level	
**Study design**	
Study population	

	Sample size
	Data collection methods
**Outcomes**	
	Stigma types mentioned
	Stigma definitions used
	Stigma scales used
	Characteristics of the scales used
	Validity and reliability of scales
	Accuracy parameters of the scales used
	Any other conceptualization of stigma used
	Outcomes and/or impacts of the study
	Limitations of the study
	The conclusion of the study
	Recommendations/Future directions
	Other additional data

Remarks–
