# Supplementary material for: The stigma associated with cutaneous leishmaniasis (CL) and mucocutaneous leishmaniasis (MCL): A protocol for a systematic review
Source: PLoS One. 2023 May 11;18(5):e0285663. doi: 10.1371/journal.pone.0285663 (PMC10174477; doi:10.1371/journal.pone.0285663)
Supplement: Attachment — Submitted filename: Response to the reviwers.docx [file pone.0285663.s004.docx]

| Reviewer #1 comments |  |
| --- | --- |
| The prevalence of MCL and CL leishmaniasis is high in South America and a lot of articles from that area are in Portuguese and Spanish. Similarly a lot of articles of middle east (where CL is common) are not in English. I recommend to include articles of Spanish and Portuguese at least and use google translator to retrieve data from those articles. | We thank the reviewer for this valid comment. We accept this comment and the manuscript was changed to reflect this (line 212) |

**Response to the reviewers**

We thank all the reviewers for their constructive comments. The responses to all comments are listed below

| Reviewer #2 comments |  |
| --- | --- |
| This article is an interesting topic, but there are several comments that must be addressed: -Since cutaneous leishmaniasis has local and regional names, it is necessary to consider these names in the search strategy, including: Biskra button and Biskra nodule; Calcutta ulcer; Jericho button; Kandahar sore; Lahore sore; Oriental button; Pian bois and old World leishmaniasis. | Thank you, reviewer, for this comment. This comment is very well received. We changed the search string by adding these suggested terms (line 198-200) |
| If the articles which skin lesions caused by the PKDL and para KDL are not included, please mention it in the exclusion criteria section. | This was corrected (line 222) |
| If the authors will be used MeSH (Medical Subject Headings) database, please declare it in the text of the protocol. | We are not using MeSH databases in this review hence we did not mention about the MeSH database |
| The year and month of the start and end of the search are not clear in the article. | This was rewritten to convey the idea more comprehensively (line 185-186) |
| There is no PRISMA flowchart in the article. | PRISMA-P Checklist was submitted as a supporting document (S1 Checklist) as per journal requirements |
| The Discussion section is very small and the limitations of this protocol are not known. | The discussion section was improved and limitations were discussed as the reviewer proposed. Line- 302-313 |
| Please state that have consulted with experts in this field to select keywords and syntax? | Thank you for this comment. This was included in the manuscript (line 189-190) |
| Please clarify the contact way with the corresponding authors, if there is missing information | This was modified in the text 219-221 |
| How do you justify the heterogeneity of included studies? | This was included line 262-268 |
| In the section on Risk of bias, please mention the quality appraisal tool in detail (scoring,…). | This was corrected (Line 277-286) |
